# Flexor Tendon Sheath Ganglion Causing Concurrent Trigger Wrist and Carpal Tunnel Syndrome

**DOI:** 10.1155/cro/8963799

**Published:** 2026-05-31

**Authors:** Mohamed Alsharif, Abdulaziz Mhannayeh, Abdullah Jabri, Bader Taftafa, Raghad Alsharif, Rama Summak, Moustafa Saeed, Abdulaziz Al-Sharif

**Affiliations:** ^1^ College of Medicine, Alfaisal University, Riyadh, Saudi Arabia, alfaisal.edu; ^2^ College of Medicine, Almaarefa University, Riyadh, Saudi Arabia, um.edu.sa; ^3^ Department of Dermatology and Venereology, Aleppo University Hospital, Aleppo, Syria, auh-orl.com; ^4^ Orthopedics Department, Kingdom Hospital, Riyadh, Saudi Arabia

**Keywords:** carpal tunnel syndrome, flexor tendon sheath, ganglion cyst, surgical excision, trigger wrist

## Abstract

**Introduction:**

Trigger wrist is a rare condition often misdiagnosed as trigger finger or carpal tunnel syndrome (CTS). It is characterized by painful clicking, snapping, or locking of the wrist during movement and can result from space‐occupying lesions, such as ganglion cysts. This case highlights an unusual presentation of trigger wrist associated with CTS caused by a ganglion cyst in the flexor tendon sheath.

**Case Presentation:**

A 44‐year‐old male presented with paresthesia, severe pain, and triggering of the right wrist. Imaging and histopathology confirmed a ganglion cyst between the flexor digitorum profundus tendons of the index and middle fingers, compressing the median nerve and causing CTS. Surgical excision via a carpal tunnel approach resulted in complete symptom resolution.

**Conclusion:**

This case emphasizes the diagnostic challenges of trigger wrist and the importance of considering it in patients with CTS‐like symptoms and mechanical wrist dysfunction. Ganglion cysts in flexor tendon sheaths are rare, and surgical excision remains an effective treatment. Increased awareness and reporting of such cases can improve clinical recognition and management.

## 1. Introduction

Trigger wrist is a rare condition characterized by painful clicking, snapping, or locking of the wrist during finger or wrist movement, often associated with carpal tunnel syndrome (CTS) [1]. Unlike trigger finger—one of the most common hand pathologies—trigger wrist is frequently misdiagnosed, leading to delayed treatment or unnecessary interventions such as A1 pulley release or steroid injections [2, 3]. The etiology of trigger wrist is diverse, with causes ranging from space‐occupying lesions (e.g., ganglions, lipomas, and giant cell tumors) to anomalous muscle bellies or tendon adhesions [4, 5].

Suematsu et al. [5] classified trigger wrist into three types: Type A (tumors/nodules in the flexor tendon sheath), Type B (anomalous muscles), and Type C (combined tumor and muscle anomaly). Later, Al‐Qattan et al. [2] expanded this classification to include tendon injuries (Type D) and mechanical causes like postsurgical adhesions (Type E). Notably, ganglions and lipomas are among the most frequently reported masses causing trigger wrist, with ganglions exhibiting recurrence rates of 4%–40% after excision [6].

Trigger wrist is an exceptionally rare clinical entity, and no population‐based incidence or prevalence data have been established in the medical literature. Its rarity is underscored by the fact that knowledge of the condition is derived almost exclusively from isolated case reports and a limited number of small case series. The largest published series has only just 15 cases to its name over a 4‐year timeframe, whereas most reports seem to be describing a mere handful of trigger wrist cases, typically just one or two at that [4, 8]. Reviews on the topic overwhelmingly agree that trigger wrist is a very rare condition when set against trigger finger, even in the rather specialist world of orthopedic and hand surgery [1]. As a result, the actual incidence of trigger wrist probably remains hidden from view due to just how uncommon it is and the tiny number of documented cases that exist across the whole world.

This case report describes a rare presentation of trigger wrist and CTS caused by a ganglion cyst in the flexor tendon sheath. While ganglions are common in the dorsum of the wrist, their occurrence in flexor tendon sheaths is exceedingly rare, with only a few cases documented [2, 3]. The patient′s symptoms—triggering, median nerve compression, and a palpable mass—highlight the importance of thorough clinical evaluation and imaging to avoid misdiagnosis. This case report was prepared in accordance with the CARE (CAse REport) guidelines.

## 2. Case Presentation

A 44‐year‐old male presented to a local hospital complaining of paresthesia and severe pain in the volar aspect of the right wrist with an associated triggering and a snapping sound, in addition to locking of the wrist that has been persistent for the last one and a half months. The flexor surface of the wrist was tender, limiting flexion of the palm and fingers of the right hand. There is no history of trauma. Patient′s history reveals a bariatric sleeve gastrectomy. Conservative treatment (nonsteroidal anti‐inflammatory drugs [NSAIDs]) was initiated, but the symptoms did not improve. Prior to surgical intervention, the patient underwent a trial of conservative management consisting of oral analgesics and NSAIDs over a period of approximately 1 week; however, no clinical improvement was observed. Given the persistence of symptoms, particularly the mechanical features of snapping and locking, further conservative measures such as splinting, corticosteroid injections, or physiotherapy were not pursued. The clinical presentation raised suspicion for an underlying space‐occupying lesion, prompting further evaluation with imaging.

**Figure 1:**
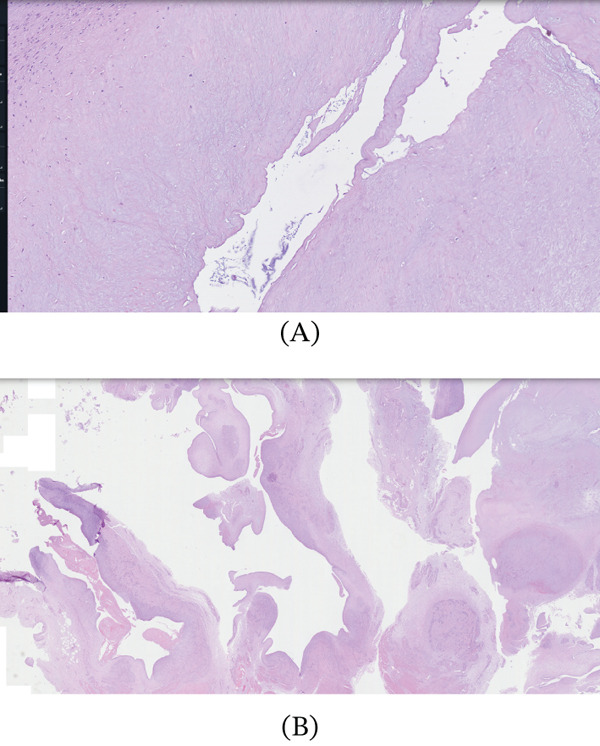
(A) Histopathological section of the excised lesion showing a ganglion cyst wall composed of dense fibrous tissue with prominent myxoid degeneration and absence of an epithelial lining (hematoxylin and eosin stain), consistent with a ganglion cyst. (B) Histopathological section demonstrating bursal tissue with granulation tissue–like vascular proliferation (red arrow), admixed with chronic inflammatory cells and fibrinoid material (blue arrow), consistent with associated chronic synovitis and bursitis (hematoxylin and eosin stain).

Differential diagnoses included all mechanical reasons of wrist flexor tendon dysfunction and space‐occupying lesions such as fibromas and giant cell tumors of the wrist. The definitive diagnosis, according to the magnetic resonance imaging (MRI) scan, surgical dissection, and histopathology, was a ganglion cyst between the flexor digitorum profundus tendons of the index and middle fingers, causing trigger wrist and CTS. Surgical exploration through a carpal tunnel approach revealed a ganglion cyst located between the flexor digitorum profundus tendons of the index and middle fingers (Figure 1A,B).

## 3. Electrodiagnostic Studies

The right median nerve showed delayed distal latency, very low amplitude compound muscle action potentials (CMAPs), low amplitude sensory nerve action potentials (SNAPs), and slow nerve conduction velocity (NCV). All these findings led to the conclusion of CTS (Table 1). These findings are consistent with moderate‐to‐severe right median neuropathy at the wrist, characterized by predominant motor axonal loss with associated sensory involvement, in keeping with chronic compressive neuropathy due to a space‐occupying lesion (trigger wrist) (Table 2).

**Table 1:** Summary of nerve conduction study findings of the median nerves, including motor and sensory parameters, reference ranges, and electrophysiological interpretation.

Parameter	Right median nerve	Left median nerve	Reference range^*^	Interpretation
Distal motor latency (DML)	Prolonged (qualitative)	Normal	≤ 4.0–4.4 ms	Conduction delay (compression‐related)
CMAP amplitude	Markedly reduced	Not reported	≥ 4–5 mV	Motor axonal loss (predominant)
Distal sensory latency (DSL)	Prolonged (implied)	Normal	≤ 3.5 ms	Sensory involvement
SNAP amplitude	Reduced	Not reported	≥ 10–20 *μ*V	Sensory axonal involvement
Sensory NCV (SNCV)	Slowed	Not reported	≥ 50–55 m/s	Secondary conduction slowing

^*^Reference ranges represent commonly accepted normal values for adult median nerve conduction studies and may vary slightly depending on laboratory standards and electrophysiological techniques.

**Table 2:** Chronological timeline of the patient′s clinical course.

Date	Clinical event
December 1, 2021	Onset of right wrist pain associated with snapping and mechanical triggering
December 11, 2021	First clinical presentation; symptoms present for approximately 6 weeks
December 12, 2021	Wrist x‐ray performed—No bony abnormality detected
December 13–20, 2021	Trial of conservative management (analgesics and NSAIDs) with no clinical improvement
December 22, 2021	Follow‐up visit due to persistent symptoms; MRI requested
December 26, 2021	MRI performed, demonstrating a partially cystic lesion within the carpal tunnel
January 20, 2022	Continued symptoms despite conservative treatment
February 16, 2022	Nerve conduction study confirming carpal tunnel syndrome
March 24, 2022	Surgical intervention: Carpal tunnel exploration and excision of the lesion
March 25, 2022	Discharged in good condition
~6 weeks postoperatively	Complete resolution of symptoms

## 4. Imaging

**Figure 2:**
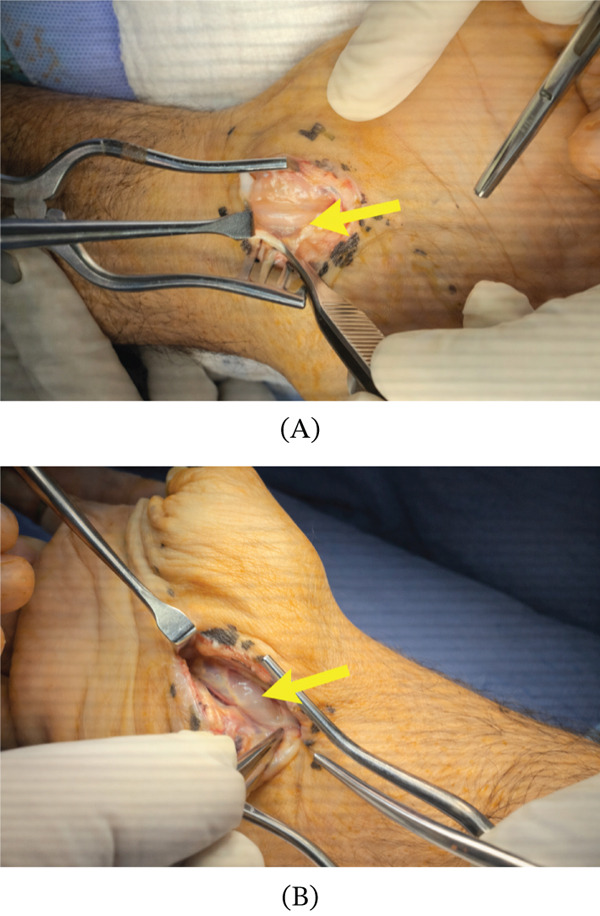
(A) Intraoperative photograph showing exposure of the volar wrist structures via a carpal tunnel approach. The ganglion cyst is identified between the flexor digitorum profundus tendons (arrow). (B) Closer intraoperative view of the ganglion cyst during surgical dissection, confirming its location between the flexor tendons (arrow).

**Figure 3:**
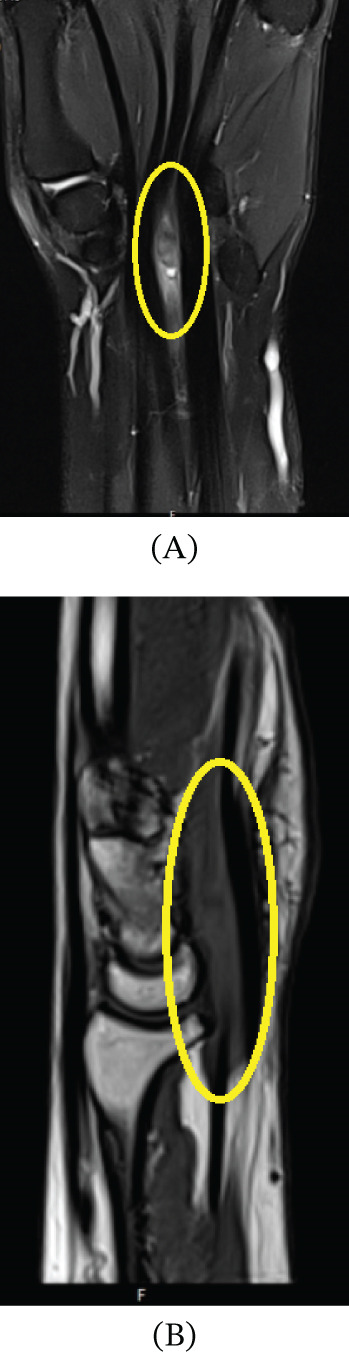
(A) Coronal noncontrast T2‐weighted fat‐suppressed MRI of the right wrist. An elongated, well‐defined oval lesion (yellow oval), measuring approximately 2.3 × 0.6 cm, is seen along the flexor tendon compartment, adjacent to the flexor digitorum profundus tendons of the index and middle fingers. The lesion demonstrates homogeneously high signal intensity, consistent with a cystic/fluid‐containing structure. Orientation: Proximal (forearm) is at the top, distal (hand) at the bottom, radial (thumb) side is on the left, and ulnar (little finger) side is on the right. (B) Sagittal noncontrast T2‐weighted fat‐suppressed MRI of the right wrist. The previously described elongated lesion (yellow oval) is visualized in longitudinal orientation along the volar aspect of the wrist, measuring approximately 2.3 × 0.6 cm. It demonstrates homogeneously high signal intensity, consistent with a fluid‐containing (cystic) structure along the flexor tendon compartment. Orientation: Proximal (forearm) is at the top, distal (hand) at the bottom, volar (palmar) aspect is anterior (toward the right side of the image), and dorsal aspect is posterior (toward the left side).

An x‐ray revealed no bony pathology; hence, a noncontrast MRI scan was performed and revealed two small cysts, one between the scaphotriquetral ligament and capitate bone, measuring 0.3 × 0.2 cm, and the second was located between the extensor digitorum tendon slips and the posterior surface of the scaphotriquetral ligament, measuring 0.4 × 0.2 cm. An oval‐shaped soft tissue lesion that was a complex cystic lesion measuring 2.3 × 0.6 cm was also noted between the flexor digitorum profundus tendons of the index and middle fingers (Figure 2A,B). The lesion showed focal cystic changes suggestive of query fibrosis. The oval‐shaped, complex cystic lesion identified on MRI between the flexor digitorum profundus tendons of the index and middle fingers correlated precisely with the intraoperative findings. During surgical exploration through a carpal tunnel approach, a ganglion cyst was visualized in the same anatomical location, confirming the MRI‐detected lesion as the source of both mechanical triggering and median nerve compression (Figure 3).

Although MRI demonstrated the presence of additional small cystic lesions in the wrist, there was no clinical, laboratory, or radiological evidence suggestive of rheumatoid arthritis or cystic ganglionosis. The patient had no history of inflammatory arthritis, no systemic symptoms, and no imaging features consistent with diffuse synovial disease. The smaller cystic lesions were considered incidental findings, while the dominant ganglion cyst arising from the flexor tendon sheath between the flexor digitorum profundus tendons of the index and middle fingers was identified as the sole pathological lesion responsible for the mechanical triggering and median nerve compression.

## 5. Histopathology

Histopathology of the specimen showed a yellowish‐white to light brown colored cut section. Microscopic features showed fibroadipose tissue with cyst‐like spaces surrounded by fibrous tissue and unremarkable striated muscle bundles. There were villous projections into the cyst‐like spaces with focal myxoid areas and occasional bland spindle cells, in addition to granulation tissue with many small blood vessels, admixed with chronic inflammatory infiltrate and fibrin. Dense connective tissue consistent with a tendon is present focally. All these features are consistent with a ganglion cyst, accompanied by chronic synovitis and bursitis. There was no nuclear atypia, and no mitotic activity was appreciated, indicating a benign lesion.

## 6. Treatment and Hospital Course

The patient was taken to the operating theater and placed supine on the operative table under general anesthesia. A tourniquet was applied to the right upper extremity; the arm was elevated for 3 min for exsanguination before the tourniquet was inflated to 250 mmHg. The right forearm was fully supinated, and the operative field was prepared and draped in the standard sterile fashion.

A 3 cm longitudinal skin incision was made at the distal wrist crease, positioned just ulnar to and parallel with the thenar crease. Dissection was carried through the subcutaneous tissue under 2.5× loupe magnification using a sharp technique down to the superficial palmar fascia, which was divided longitudinally. The transverse carpal ligament was then exposed; its proximal edge was identified at the level of the distal palmar crease and incised longitudinally with a scalpel in line with the ring finger ray, medial to the thenar muscles. The median nerve was identified, and a blunt dissector was passed between the nerve and the distal aspect of the transverse carpal ligament, which was then fully divided, completing the carpal tunnel release. The antebrachial fascia was additionally divided for 2 cm proximal to the wrist crease along the ring finger axis to ensure adequate decompression.

Following decompression of the carpal tunnel, the volar aspect of the right wrist was systematically explored. An oval soft tissue neoplasm measuring approximately 1 × 0.75 cm was identified within the carpal tunnel, situated along the third long flexor capsula between the flexor digitorum profundus tendons of the index and middle fingers, lying just underneath the median nerve. The lesion corresponded precisely to the complex cystic mass identified on the preoperative MRI. The ganglion cyst, along with its stalk, was radically excised in its entirety and submitted for histopathological analysis. No synovectomy was required, as there was no macroscopic evidence of synovial inflammation. To minimize the risk of recurrence, the cyst was excised with its stalk and base attachment to the flexor tendon sheath; care was taken to achieve complete removal without rupture of the cyst wall.

The wound was irrigated thoroughly, and hemostasis was secured. The tourniquet was deflated, and intraoperative assessment confirmed full range of motion of all fingers and tendons under direct vision as well as complete release of the median nerve. The skin was closed in a subcuticular fashion. A sterile dressing and elastic bandage were applied. There were no intraoperative complications.

Postoperatively, the patient demonstrated complete clinical recovery. At follow‐up, there was full resolution of wrist pain, mechanical triggering, and median nerve–related paresthesia, with return to normal hand and wrist function. No residual weakness, sensory deficits, or recurrence of symptoms was noted. These findings further support surgical excision of the causative lesion as a definitive and effective treatment for trigger wrist associated with space‐occupying lesions, particularly when accompanied by CTS.

## 7. Discussion

This case highlights the diagnostic complexity of trigger wrist, a rare clinical entity often mistaken for trigger finger or idiopathic CTS due to overlapping symptomatology. The patient had symptoms of median nerve compression and triggering during wrist flexion and extension, which is consistent with other reports in which space‐occupying lesions were linked to two different pathologies [8–10]. MRI and histopathological analysis confirmed a ganglion cyst compressing the median nerve and causing mechanical triggering, consistent with Type A of Suematsu′s classification of trigger wrist etiologies [5]. The concordance between preoperative MRI findings and intraoperative observations strengthened diagnostic confidence, as the lesion′s location, morphology, and relationship to the flexor tendons and median nerve were consistent across imaging and surgical exploration.

The risk of misdiagnosis is substantial. Unlike trigger finger, trigger wrist does not involve tenderness over the A1 pulley, and symptoms are typically elicited at the wrist rather than the digit [1]. Failure to recognize this distinction can delay accurate diagnosis and appropriate intervention. A thorough understanding of the distinguishing clinical, anatomical, and diagnostic features between trigger finger and trigger wrist is therefore essential to avoid delays in management and prevent unnecessary procedures [1, 4].

Trigger finger (stenosing tenosynovitis) involves narrowing of the A1 pulley at the metacarpophalangeal joint, causing the flexor tendon to catch or lock as it passes through, producing a characteristic snap or trigger at the base of the digit [9]. It typically presents with a tender nodule over the A1 pulley, pain and stiffness in the finger, and triggering or locking of the affected digit in flexion. The condition is most common in middle‐aged women and is associated with diabetes mellitus, rheumatoid arthritis, and repetitive hand use [11. Diagnosis is largely clinical, and conservative management with corticosteroid injections or A1 pulley release is often curative [9].

In contrast, trigger wrist is far rarer and involves mechanical obstruction within the carpal tunnel or flexor tendon sheath at the wrist level, typically due to space‐occupying lesions, anomalous muscles, or tendon adhesions [2, 5]. Symptoms manifest as painful snapping, clicking, or locking of the wrist during flexion or extension and may be accompanied by signs of median nerve compression, such as paresthesia or thenar weakness when the carpal tunnel is involved. Crucially, the A1 pulley is nontender, and digital triggering is absent, which serves as a key differentiating feature from trigger finger. Diagnosis requires imaging (MRI or ultrasound) to identify the underlying lesion, and treatment is surgical excision rather than pulley release [4, 12]. These distinguishing features are summarized in Table 3.

**Table 3:** Comparative features of trigger finger and trigger wrist to aid differential diagnosis.

Feature	Trigger finger	Trigger wrist
Site of pathology	A1 pulley at the metacarpophalangeal joint	Carpal tunnel/flexor tendon sheath at the wrist
Triggering location	Digit (finger or thumb)	Wrist (during wrist/finger motion)
A1 pulley tenderness	Present (hallmark finding)	Absent (key differentiator)
Palpable nodule	Often present at the A1 pulley (MCP joint)	May be present at the wrist (e.g., ganglion cyst)
Nerve involvement	Typically absent	Median nerve compression (CTS) may coexist
Common etiology	Stenosing tenosynovitis of the A1 pulley	Space‐occupying lesion (ganglion, lipoma, and giant cell tumor), anomalous muscle, and tendon adhesion
Associated conditions	Diabetes mellitus, rheumatoid arthritis, and repetitive hand use	Carpal tunnel syndrome, prior wrist surgery, or trauma
Key diagnostic investigation	Primarily clinical; x‐ray to exclude bony pathology	MRI or ultrasound to identify underlying mass; nerve conduction studies if CTS suspected
First‐line treatment	Corticosteroid injection or A1 pulley release	Surgical excision of causative lesion ± carpal tunnel release
Incidence	Common (one of the most frequent hand conditions)	Rare (documented primarily through isolated case reports)

*Note:* Sources: Suematsu et al. [5], Al‐Qattan et al. [2], Park et al. [4], Lychagin et al. [8], Makkouk et al. (2008), Jeanmonod et al. (2024), and Arumugam et al. [1].

Abbreviations: CTS, carpal tunnel syndrome; MCP, metacarpophalangeal; MRI, magnetic resonance imaging.

While MRI plays a pivotal role in preoperative planning, it may fail to detect small lesions or rare anomalies such as accessory muscles [12]. In such cases, intraoperative exploration becomes both diagnostic and therapeutic. This underlines the need for maintaining a high index of suspicion, especially when imaging is inconclusive, but clinical features persist.

Moreover, for patients displaying symptoms of median nerve compression, such as pain and paresthesia, nerve conduction studies are imperative [2].

Surgical excision of the ganglion cyst, combined with carpal tunnel release, led to immediate symptom resolution in our patient. This outcome aligns with previously reported cases, such as those by Imai et al. [13] and Al‐Qattan et al. [2], where similar interventions yielded favorable results. However, ganglion recurrence remains a significant concern, with revision cases showing recurrence rates as high as 15% [6]. Long‐term follow‐up is therefore essential to monitor for potential recurrence or residual nerve dysfunction.

Although surgical excision remains the most effective way of treating trigger wrist, other strategies have been described. Wrist splinting, rest, and NSAIDs may provide temporary relief in mild or early cases but rarely provide lasting resolution [1]. Ultrasound‐guided aspiration of ganglion cysts has been attempted, though recurrence rates of up to 40%–50% limit its long‐term success [6]. Corticosteroid injections and observation are options if the lesion is small or is inflammatory, while endoscopic approaches can reduce scarring [8]. Nonetheless, surgical resection remains the gold standard to ensure definitive treatment and prevent recurrence.

Our findings are consistent with ganglion‐associated cases reported by Altaf et al. [3] and Al‐Qattan et al. [2], wherein cystic lesions caused mechanical triggering and median nerve compression. In contrast to reports involving anomalous muscles such as hypertrophic flexor digitorum superficialis bellies [14], our patient′s lesion was purely cystic, which was confirmed histopathologically.

Clinicians should include trigger wrist in the differential diagnosis of patients presenting with CTS‐like symptoms and mechanical triggering, especially when the A1 pulley is nontender and symptoms originate from the wrist [1]. Imaging modalities, particularly ultrasound and MRI, are instrumental for identifying soft tissue masses, though they may not detect all lesions [12]. When imaging is inconclusive, surgical exploration should be considered. Importantly, complete excision of the cyst and its stalk is crucial to reduce recurrence risk [6].

## 8. Conclusion

Trigger wrist is a very uncommon pathology, especially in comparison to the most common hand pathology, which is trigger finger. This is why trigger wrist should always be in physicians′ lists of differential diagnoses, and cases of trigger wrist should be reported to further enrich the literature to better aid physicians in diagnosis and treatment. In addition, a ganglion cyst in a flexor tendon sheath is also very uncommon, with a few cases reported in the literature, which makes our patient′s presentation even more noteworthy.

## Funding

No funding was received for this manuscript.

## Ethics Statement

Institutional Review Board (IRB) approval for publication of this case report was obtained from the relevant ethics committee at the reporting institution. Written informed consent for publication of the clinical details and accompanying images was obtained from the patient prior to manuscript submission.

## Conflicts of Interest

The authors declare no conflicts of interest.

## Data Availability

Data sharing is not applicable to this article as no datasets were generated or analyzed during the current study.
